# Circles within circles: crosstalk between protein Ser/Thr/Tyr-phosphorylation and Met oxidation

**DOI:** 10.1186/1471-2105-14-S14-S14

**Published:** 2013-10-09

**Authors:** R Shyama Prasad Rao, Dong Xu, Jay J Thelen, Ján A Miernyk

**Affiliations:** 1 Department of Biochemistry, University of Missouri, Columbia, MO 65211, USA; 2 Interdisciplinary Plant Group, University of Missouri, Columbia, MO 65211, USA; 3 Computer Science, University of Missouri, Columbia, MO 65211, USA; 4 Plant Genetics Research Unit, USDA, Agricultural Research Service, University of Missouri, Columbia, MO 65211, USA

**Keywords:** Methionine, methionine sulfoxide, oxidation, protein phosphorylation, regulation, reversible posttranslational modifications, signalling

## Abstract

**Background:**

Reversible posttranslational protein modifications such as phosphorylation of Ser/Thr/Tyr and Met oxidation are critical for both metabolic regulation and cellular signalling. Although these modifications are typically studied individually, herein we describe the potential for cross-talk and hierarchical regulation.

**Results:**

The proximity of Met to Ser/Thr/Tyr within the proteome has not previously been addressed. In order to consider the possibility of a generalized interaction, we performed a trans-kingdom sequence analysis of known phosphorylation sites in proteins from bacteria, fungi, plants, and animals. The proportion of phosphorylation sites that include a Met within a 13-residue window centered upon Ser/Thr/Tyr is significantly less than the occurrence of Met in proximity to all Ser/Thr/Tyr residues. Met residues are present at all positions (-6 to +6, inclusive) within the 13-residue window that we have considered. Detailed analysis of sequences from eight disparate plant taxa revealed that many conserved phosphorylation sites have a Met residue in the proximity. Results from GO enrichment analysis indicated that the potential for phosphorylation and Met oxidation crosstalk is most prevalent in kinases and proteins involved in signalling.

**Conclusion:**

The large proportion of known phosphorylation sites with Met in the proximity fulfils the necessary condition for cross-talk. Kinases/signalling proteins are enriched for Met around phosphorylation sites. These proteins/sites are likely candidates for cross-talk between oxidative signalling and reversible phosphorylation.

## Background

The proteome includes the combination of at least three components; genome-encoded proteins, the products of alternative initiation and splicing, and of posttranslational modifications (PTM). This combination is orders of magnitude larger than could be solely genome-encoded [1]. The diversity of PTM is extraordinarily large; to date as many as 435 different PTMs are known [2-5]. They can be reversible (e.g., acetylation) or irreversible (e.g., proteolytic cleavage), enzyme catalysed (e.g., kinase-mediated phosphorylation) or the result direct chemical reaction (e.g., oxidation), and individual or combinatorial [6,7]. *In toto*, these factors comprise a well-adapted basis for signalling, regulation, targeting, and interaction, all at least potentially in the absence of *de novo *protein synthesis [3,8].

Among PTM, reversible phosphorylation has been the most extensively studied [9-11]. This is in part because reversible phosphorylation is a component of both cellular signalling [8,12] and direct regulatory control of protein function [13-15]. Protein phosphorylation can be stoichiometric at an individual site, or combinatorial and sequential/hierarchical at multiple sites [16,17]. During the 'genomic era,' an enormous number of different protein kinases and phospho-protein (P-protein) phosphatases were identified, and reversible protein phosphorylation has been shown to be involved in regulating many fundamental cellular processes. It has been reported that the combination of genes encoding protein kinases and P-protein phosphatases constitute between 2 and 4% of the total number of genes in a typical eukaryotic genome [18]. *Saccharomyces cerevisiae *has genes for 113 protein kinases while the *Homo sapiens *has 518 such genes [19]. The genome of the reference dicot plant *Arabidopsis thaliana *includes genes encoding 1055 protein kinases [19] and 112 P-protein phosphatase catalytic subunits [20]. Clearly, this PTM is ubiquitous across all branches of the tree of life [21].

Reactive oxygen species (ROS) are an inevitable consequence of aerobic metabolism [22-24]. High levels of ROS cause oxidative damage to proteins, which is lethal unless repaired or reversed [25,26]. In contrast, low levels of ROS have evolved functional roles in many aspects of cellular signalling [27-29]. The reversible chemical oxidation of Met to methionine sulfoxide (MetSO) spans the two extremes in response to ROS. Oxidation of Met to MetSO can damage/inactivate proteins, but because this PTM is enzymatically reversible [30,31] it can participate in the types of regulation [32] and signalling [33,34] typified by reversible phosphorylation. Met oxidation is a particularly important component of cellular responses to oxidative stress [35,36].

In addition to Ser/Thr/Tyr phosphorylation and Met oxidation, other common reversible PTMs include Ser/Thr O-glycosylation [37], Lys/Arg methylation [38], and Lys acetylation [39]. There are examples of each directly regulating protein activities as well as playing roles in cellular signalling [40-43]. While a single PTM can modulate protein function, combinatorial and sequential/hierarchical interplay among two or more types of PTM can integrate signals from multiple pathways [6,44,45]. Dynamic interactions among PTM can take place at the same target residue [5,46], or there can be cross-talk between or among multiple residues [47,48]. In some instances the PTM of a specific residue can require a prior (primed) PTM at another site [6,17,49].

It was recently suggested that reversible Met oxidation might serve as a rheostat to control proximal Ser/Thr/Tyr phosphorylation [50,51], and that this could be a mechanism for integrating information from both metabolic and ROS-based signalling pathways [52,53]. However, it is not known how often phosphorylation sites contain Met residues in their proximity. It is important to identify candidate proteins with coupled Met oxidation and phosphorylation sites. The results described herein are from a study undertaken to test the possibility that phosphorylation and Met oxidation crosstalk has a broad occurrence.

## Methods

### Proteome sequences and phosphorylation sites

In order to consider our analysis of potential crosstalk in as broad a context as possible we have employed a data-mining strategy. Selected reference proteome or complete proteome sequences were downloaded from Uniprot [54], NCBI [55], or TAIR [56]. Experimentally confirmed phosphorylation (Ser/Thr/Tyr) site information was obtained from multiple databases [21,57]. Plant-specific phosphorylation site data was obtained from P^3^DB [58]. Our aim was to explore the proportion of phosphorylated (and potentially phosphorylatable) sites that contain a Met residue within 13 residue window in different taxa. We used sequences and sites from several species of plants, yeast, animals, bacteria, and archaea (Additional file 1). Because Met oxidation is an aerobic process, we also included species that have an anaerobic metabolism/lifestyle such as *Bacteroides fragilis*, *Clostridium botulinum*, and *Ascaris suum*.

### Phosphorylation site predictions

Phosphorylation sites were predicted for complete proteomes using Musite [59], NetPhosBac [60], or Disphos [61]. Predictions were based on taxa-specific Ser/Thr/Tyr models at 95% specificity for the respective proteomes. Predictions were made to compare the differences, if any, between the proportion of Met residues in the vicinity of experimentally known phosphorylation sites and predicted phosphorylation sites.

### Sequence analyses

The proportion of Ser/Thr/Tyr sites that include at least one Met-residue in a window ranging up to 21-residues was scanned. As most known phosphorylation motifs are less than 13-residues in length [62], a 13-residue window (six residues either side of the phosphorylation site) was selected for our analyses. Amino acid frequencies for individual proteomes were computed. Amino-terminal Met-residues were discounted in both calculations of Met frequency and sequence-distribution analyses. The hydropathy score for each site (Ser/Thr/Tyr centered in a 13-residue window; the phosphorylation site itself was not included) was calculated using the Kyte-Doolittle scale [57,63].

In order to evaluate the extent of conservation of phosphorylation sites that contain a Met, and to identify candidate proteins for Phos-Met oxidation crosstalk in plants, a PSI-BLAST search was conducted for *Arabidopsis thaliana, Glycine max*, and *Oryza sativa *proteins that include a Met-residue within the 13-residue phosphorylation-site window. Proteins from these three taxa account for ~90% of known phosphorylation sites in plants. The results were used to identify related sequences in five additional proteomes (*Pinus taeda*, *Selaginella moellendorffii*, *Physcomitrella patens*, *Chlamydomonas reinhardtii*, and *Cyanidioschyzon merolae*), using a significance E-value cutoff of 0.00001. The total number of phosphorylation sites with Met-conservation was then calculated among the eight disparate taxa. Additionally, the proportion of hydrophobic residues (Met/Leu/Ile/Val/Phe/) was enumerated for each phosphorylation site.

### Gene ontology enrichment analysis

A gene ontology (GO) term-enrichment analysis was performed for proteins that contain at least one known phosphorylation site including a Met within our specified ± 6-residue window, using the agriGO webserver [64] for *A. thaliana *or the g:Profiler webserver [65] for *S. cerevisiae *and *H. sapiens*. A hypergeometric test (with Bonferroni correction for multiple comparisons) was used to select significantly enriched (p < 0.05) terms [64,65]. A log_2 _ratio of numbers of each significantly enriched GO term in the test set (phosphoproteins that have Met residues in the vicinity of phosphorylation site) to background set (all phosphoproteins) was calculated. All data analyses were performed using Python ver. 2.7. A Z-test was used to calculate the difference between two group proportions; p < 0.05 was considered significant.

## Results

**Figure 1:**
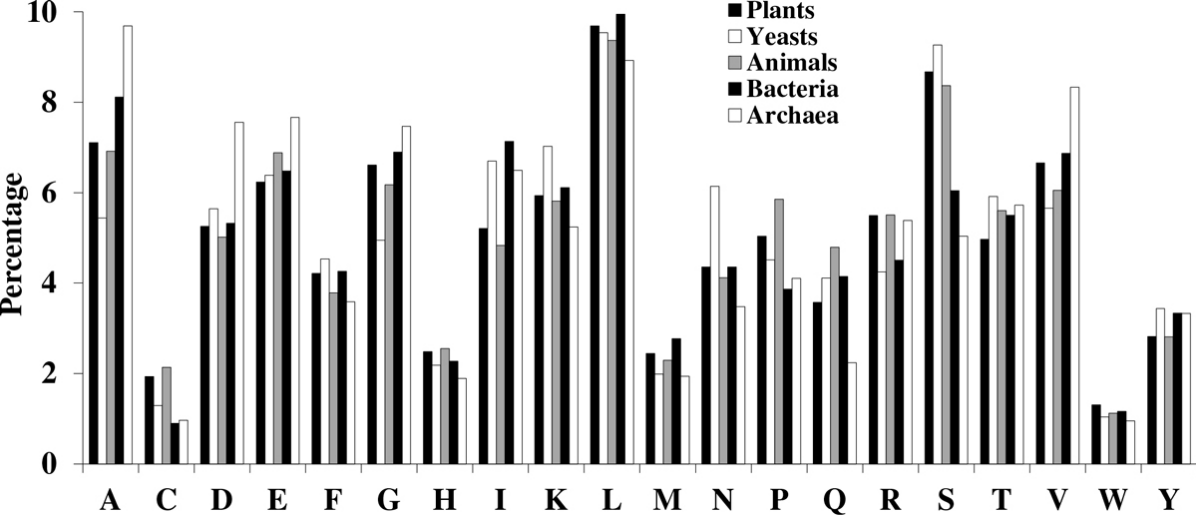
**Proportions (%) of amino acids present in the translated proteomes from different taxa**. Met is the least abundant hydrophobic residue. Proportions of Ser and Thr are nearly equal in bacteria and archaea.

Evaluating the translated proteome from diverse taxa, it is clear that Met belongs to the group of relatively low-abundance amino acids along with Cys, His, Trp, and Tyr (Figure 1). In plants, yeast, and animals Ser has a relatively high abundance, and Thr is of intermediate abundance in all taxa. The low Met abundance is in contrast to the other members of the hydrophobic group (Leu, Ile, Val, and Phe) all of which are moderately to highly abundant.

**Figure 2:**
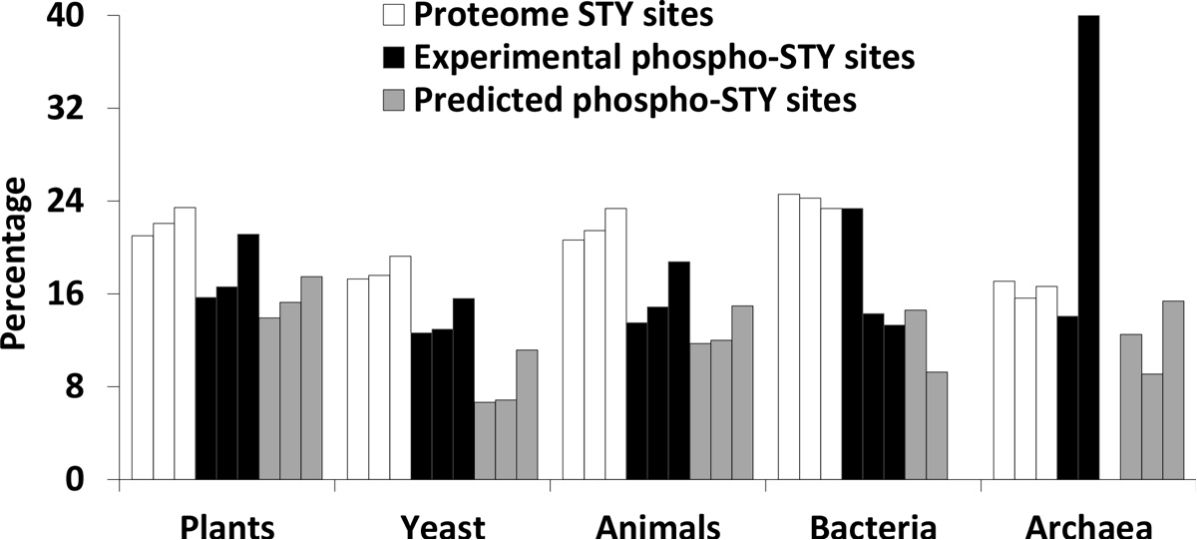
**The percentage of Ser/Thr/Tyr sites (individually from left to right in each group) including a Met residue within ± 6 window in different taxa**. On average, phosphorylated sites contain less Met compared to all Ser/Thr/Tyr sites in proteomes. In eukaryotes, Tyr sites contain proportionally more Met compared to Ser and Thr sites. The large deviation from the otherwise 'normal' pattern seen with bacteria and archaea is likely due to the small number of phosphorylated sites.

The proportion of Ser/Thr/Tyr sites which include a Met within the window of ± 6 residues is shown in Figure 2. On average 14% of phosphorylated sites contained a Met. Overall, the proportions of phosphorylated Ser/Thr/Tyr sites (experimentally determined and predicted) including a Met was significantly less (p ~ 0, Z-test for two proportions) compared to proteome-wide Ser/Thr/Tyr sites. Proportionally, Tyr sites contain more Met than Ser and Thr sites in eukaryotes. The apparent deviation from this pattern seen with archaeal and bacterial proteins can likely be attributed to the small total number of phosphorylated sites in these organisms. Proteome specific proportions within individual taxa are shown in Additional file 1.

**Figure 3:**
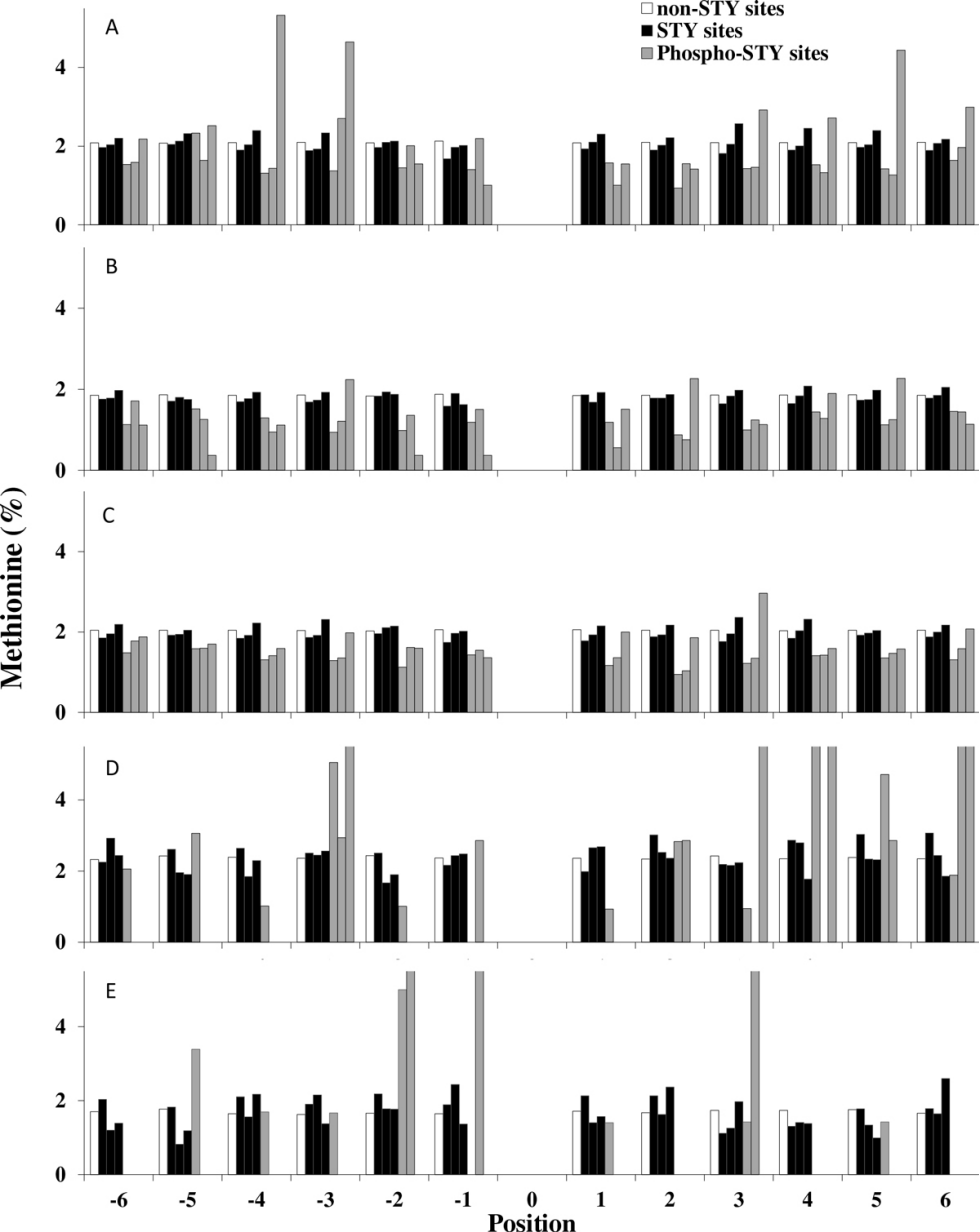
**Distribution of Met residues (%) around non-STY and STY (individually from left to right, all and phosphorylated) sites in plants (A), yeast (B), animals (C), bacteria (D) and archaea (E) (*n *= 10353, 3645, 21562, 126 and 63 phosphoproteins respectively)**.

The overall positional distribution of Met in the proteome of eukaryotic organisms is nearly uniform. In Figure 3 we have plotted the distribution of Met within the ± 6 residue window centered on; any amino acid ("non-STY"), Ser, Thr, or Tyr (STY), or P-Ser, P-Thr, or P-Tyr (Phospho-STY). The distribution of Met flanking P-Tyr residues is enriched at positions -4, -3, and +5 in plant proteins, and to a lesser degree at position +3 in all but yeast proteins. Considering the large sample size, the position-specific proportional differences are significant (p ~ 0, χ2 test for independence). In contrast, the apparent enrichment of Met at several positions in bacterial or archaeal proteins is based upon much smaller sample numbers and is not significant.

The patterns are somewhat more complex when comparing the distribution of other members of the hydrophobic amino acid homology group (Leu, Ile, Val, and Phe) within the ± 6 residue window used to evaluate Met distribution (Additional file 2). With few exceptions, plant, yeast, and animal proteins have a lower occurrence of a hydrophobic residue within the ± 6 residue window centered on a P-Ser/Thr/Tyr residue. The exceptions include yeast position +1, and mammalian positions -1 and +3 from a P-Tyr residue (Additional file 2). Overall distribution of hydrophobic residues in the ± 6 window of prokaryotic and archaeal proteins is quite uneven, but it must be considered that the total number of phosphorylation sites in these proteins is much lower than in eukaryotic proteins (e.g., *E. coli *has been reported to have only 32 P-Tyr-containing proteins and two tyrosine-kinases [66].

**Figure 4:**
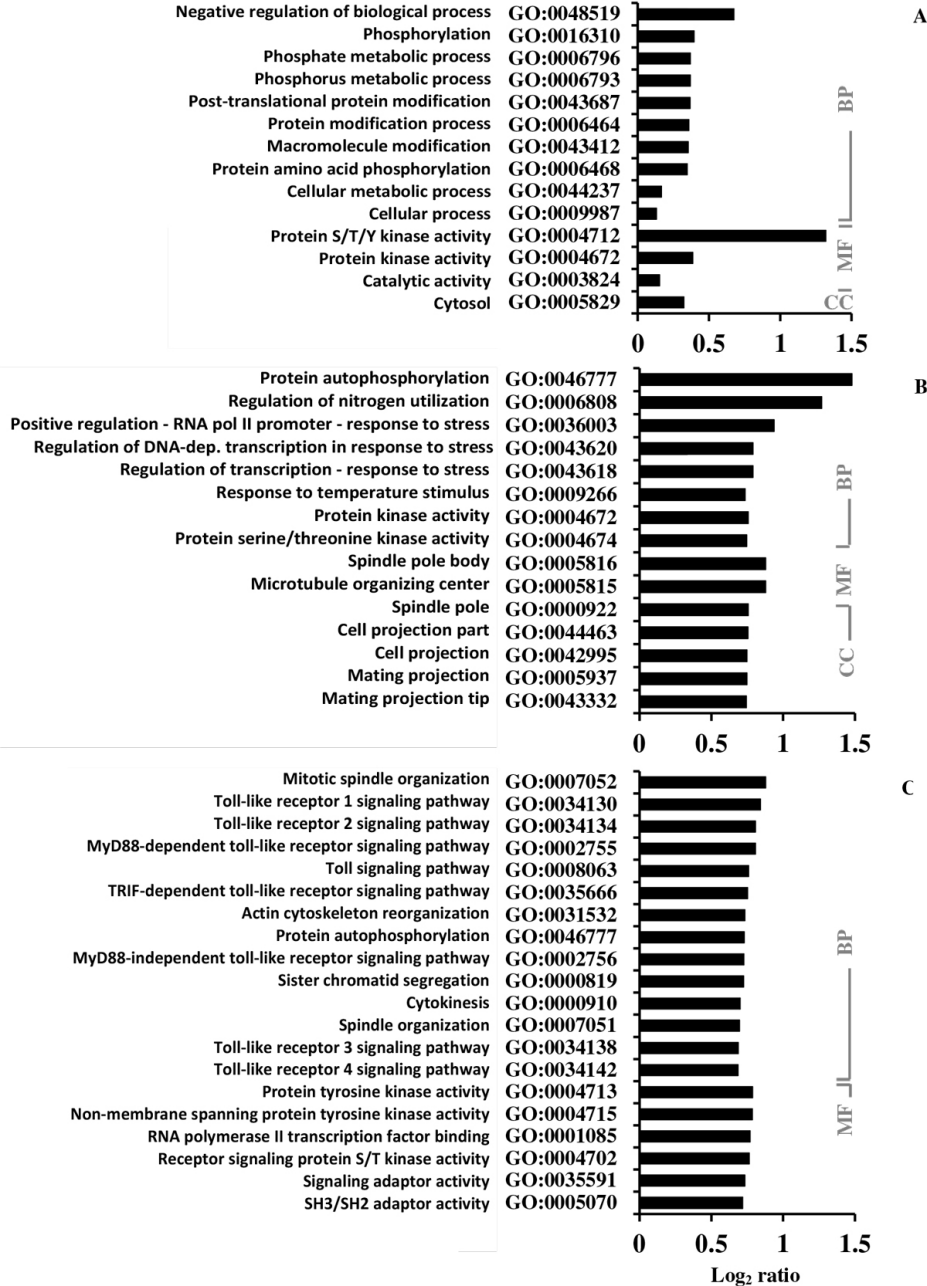
**Results from GO analyses**. The proteins (test set) that contain a Met residue within ± 6 window around known phosphorylation sites are enriched (p < 0.05, hypergeometric test and z-score > 1.96; X-axis shows the log_2 _ratio of number of each significantly enriched GO term in test set against background set) for several GO terms against the total phosphoproteins (background set) in plant (*A. thaliana*, A), yeast (*S. cerevisiae*, B) and animal (*H. sapiens*, C). In general Met oxidation and phosphorylation cross-talk are more likely in protein kinases, as several GO terms related to kinase activity as enriched in all three taxa. (BP: Biological Process, MF: Molecular Function, and CC: Cellular Component).

We conducted GO analysis of the sequences of all the plant, yeast, and animal proteins that have a Met-containing phosphorylated-site, in order to test for enrichment in functional domains/motifs. The analyses were conducted versus a total P-proteins background (Figure 4). Bacteria and archaeal proteins were not included in this analysis because of the small sample sizes. Only the GO terms that are significantly enriched (p < 0.05, hypergeometric test) in our dataset are displayed; most are related to protein kinases, phosphorylation, signalling, and regulation. There is also enrichment in proteins annotated as having an involvement in cell division in yeast or animals, but not in plants (Figure 4). For example, a log_2 _value of 0.5 indicates 41% increase in a specific GO term in the test set (proteins that contain a Met near phosphorylation site) as against the background set (all phosphoproteins).

**Figure 5:**
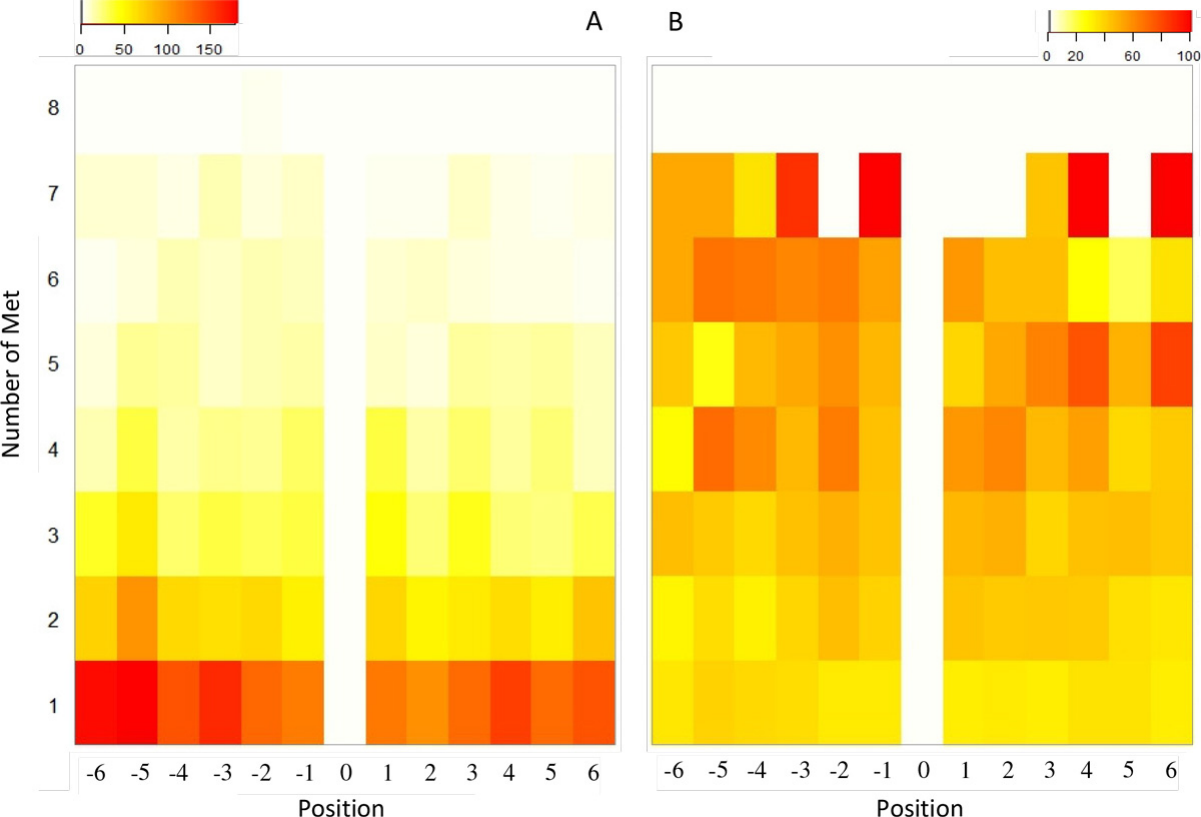
**The occurrence of Met versus other hydrophobic amino acids vicinal to phosphorylation sites**. (A) The heat map indicates the proportion of phosphorylation sites at each level of Met conservation. Many phosphorylation sites with conserved (e.g., ≥ 6/8) Met are present across different (eight) taxa. (B) Proportion (%) of sequences (in disparate taxa) including a hydrophobic (Val/Ile/Leu/Phe) residue. At higher Met conservation, a higher proportion (%) of remaining sequences includes a hydrophobic residue in place of Met.

We assume that a phosphorylation-site that includes a Met at the same position within the ± 6 residue-window among diverse taxa would have the greatest probability for regulatory crosstalk. We specifically searched proteins from 8 plant taxa in order to evaluate potential candidates. A list of the consensus 13-residue sequences for potentially conserved P-Ser/Thr/Tyr sites which include a Met residue is presented in Additional file 3. The extent of Met positional-conservation is presented as a heat map in Figure 5A. Several sites with a "high degree" of positional conservation for Met (≥ 6/8 homologs) were detected. At higher Met conservation, most of the non-Met containing remaining sequences have a hydrophobic (Val/Ile/Leu/Phe) residue at that position (Figure 5B).

**Figure 6:**
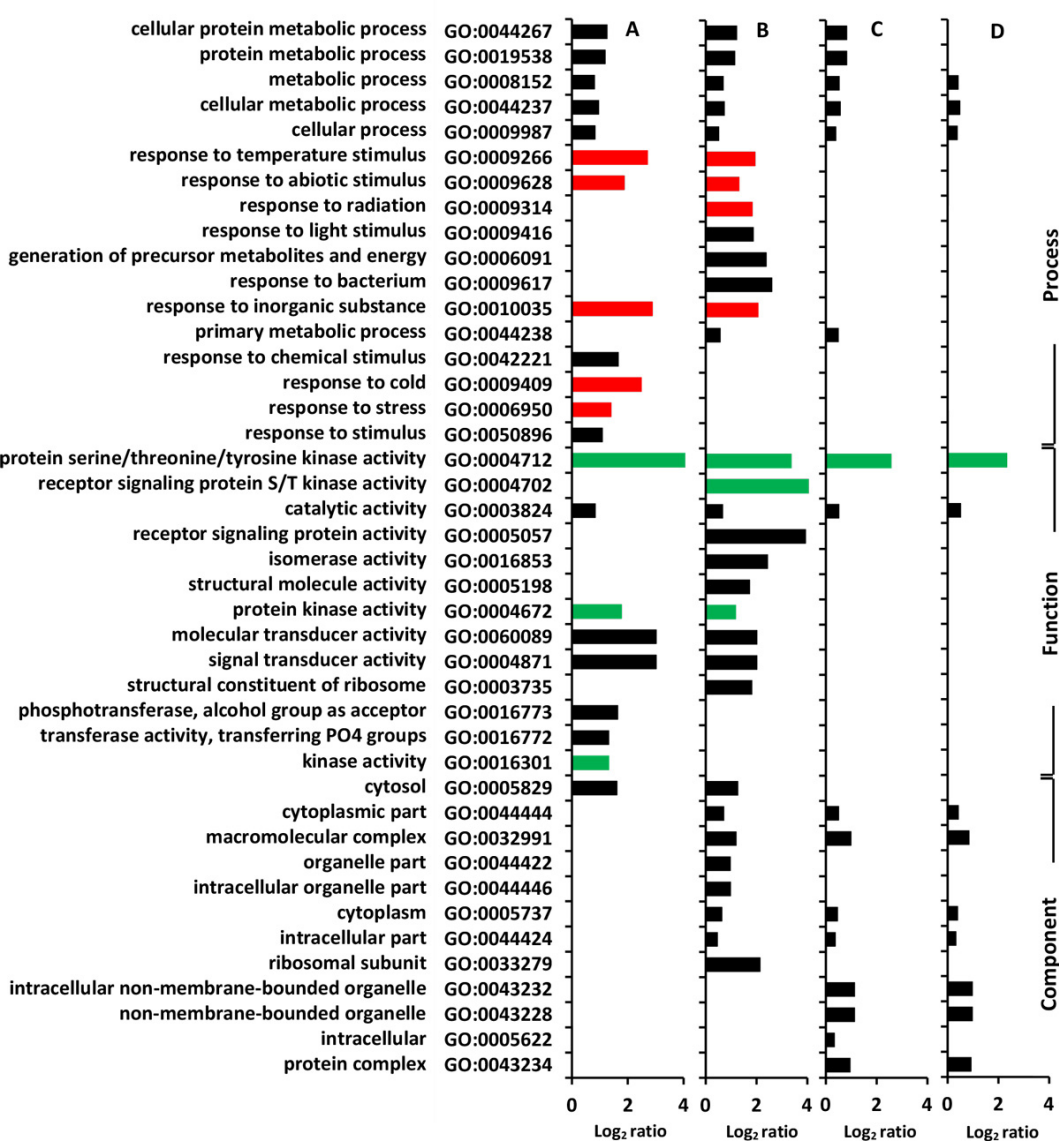
**Results from GO term enrichment analyses**. The *A. thaliana *proteins (test set) that contain a known phosphorylation site (based upon published data from *A. thaliana, G. max*, or *O. sativa*) including a Met residue within the ± 6 residue window shows significant enrichment (p < 0.005, hypergeometric test, X-axis shows the log_2 _ratio of number of each significantly enriched GO term in test set against background set) for several GO terms when the Met is conserved (panel A ≥7, B ≥6, C ≥5 and D ≥4) in eight disparate taxa. As the Met conservation level decreases the significance of the GO term enrichment decreases compared to the background of *A. thaliana *P-proteins. The GO term enrichment results agree with the proposal that Met oxidation:phosphorylation-site cross-talk is most likely in kinases (green bars) and various stress-related proteins (red bars).

The proteins that contain an experimentally verified phosphorylation site with a vicinal Met show significant enrichment (p < 0.05, hypergeometric test) for many GO terms when the Met is conserved (panel A ≥7, B ≥6, C ≥5 and D ≥4) in eight disparate plant taxa (Figure 6). Many significant GO terms are enriched several-fold; kinase activity (GO:0004712), for example. However, as the Met conservation level decreases the GO term enrichment also decreases until it is no longer significant when compared to the background of total phosphoproteins. The results from GO term analysis indicate that Met and phosphorylation site cross-talk is likely to be more prevalent in kinases and various stress-related proteins (Figure 6).

**Figure 7:**
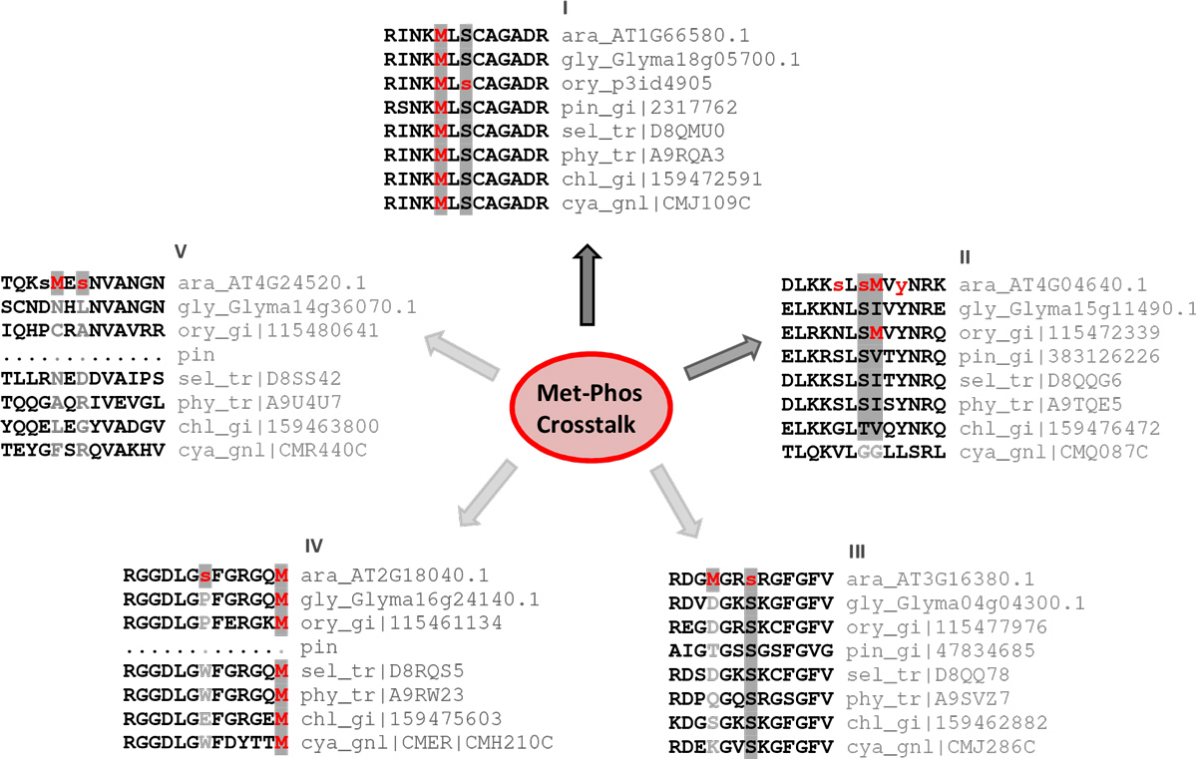
**Crosstalk between Met and Ser/Thr/Tyr might be related to the nature/extent of conservation of Met in proteins**. (I) Met could play a regulatory role (crosstalk) (for example, senescence associate protein: AT1G66580) when it is conserved across species. (II) It might play only a structural role in a protein (for example, ATPase F1º AT4G04640), present as a hydrophobic residue. (III-V) If either the phosphorylation site or the Met residue itself is not conserved, it is unlikely that crosstalk will evolve in such proteins (for example, Poly(A) binding protein: AT3G16380.1; peptidylprolyl cis/trans isomerase: AT2G18040.1; P450 reductase: AT4G24520.1). Crosstalk can also depend on the distance between phosphorylation site and Met residue in the 1° sequence, and the 3° structural context. (ara: *Arabidopsis thaliana*, gly: *Glycine max*, ory: *Oryza sativa*, pin: *Pinus Taeda*, Sel: *Selaginella moellendorffii*, phy: *Physcomitrella patens*, chl: *Chlamydomonas reinhardtii*, and cya: *Cyanidioschyzon merolae*).

Figure 7 shows some of the possible scenarios in the phosphorylation and Met-oxidation crosstalk. Depending on the extent of evolutionary conservation of Met (Figure 5, 7), it could play a regulatory role if it is well conserved across species (for example, senescence-associate protein: AT1G66580) or as a structural role if it serves merely as a hydrophobic residue along with VILF residues (for example, as in ATPase F1º: AT4G04640). The relevance of the crosstalk may also depend on the distance between the phosphorylation site and Met residue. The farther away the Met residue is, the less likely it is involved in crosstalk.

## Discussion

Two unrelated plant enzymes, cytoplasmic nitrate reductase [50], and mitochondrial pyruvate dehydrogenase [51], have a Met residue proximal to a regulatory phosphorylation site, and in both cases oxidation of this Met to MetSO inhibits phosphorylation. It has been suggested that the redox status of these Met residues allows the enzymes to monitor oxidative stress, and crosstalk between these two PTMs can serve to fine-tune activity/metabolism [52,53]. The study described herein was undertaken to evaluate how widespread the potential is for this sort of regulation by crosstalk between PTMs.

A 13-amino acid motif centered on the phosphorylatable Ser/Thr/Tyr-residue is apparently adequate for recognition by protein kinases [62,67]. Multiple distinct factors likely contributed to evolution of this motif, including the occurrence of each of the amino acids [68,69] and the constraints inherent to protein structure [70].

Given that thousands of phosphorylation sites are known in proteomes spanning taxa [57], the presence of Met in ~14% of these sites (Figure 2, Additional file 1) translates into a number of potential sites and proteins for phosphorylation and Met-oxidation crosstalk. Many phosphorylation motifs contain hydrophobic residues (Met/Val/Ile/Leu/Phe). This presumably reflects some essential aspect of protein structure or folding. However, Met is less abundant than the other hydrophobic residues [71], and is less likely to accommodate a structural requirement [72]. Furthermore, biosynthesis of Met is more energetically expensive than other members of the hydrophobic group [69,71,73]. Considering all these constraints, the presence of Met in the phosphorylation motif would need to be of some evolutionary benefit otherwise it would have been lost during subsequent optimizing of the regulatory network [74]. We propose that the benefit might have been acquisition of the ability to monitor ROS status by reversible Met oxidation, a capability not available with other hydrophobic residues [75]. Even non-surface exposed Met residues are susceptible to oxidation because of local polypeptide flexibility [76].

Met residue in the neighbourhood of phosphorylation sites can have two distinct roles (Figure 7). It could have a solely structural role in which Met acts as a hydrophobic residue in a kinase recognition motif. If Met is replaced by other hydrophobic (VILF) residues in related species, then it is likely to have a structural role. On the other hand, Met could play a regulatory role if it is conserved near phosphorylation site in a protein from diverse species [75]. We identified a large number of plant proteins and phosphorylation sites including Met that are highly conserved (Figure 5, Figure 7 and Additional file 3). These proteins are potential candidates wherein Met oxidation may be involved in coupling oxidative signals with phosphorylation and regulation of protein function.

This proposed evolutionary acquisition of Met/MetSO as a mechanism for fine-control of phosphorylation status might be addressed by comparison of phosphorylation sites in proteins from aerobic microorganisms with those from closely-related but obligate anaerobes. If Met oxidation exerts an influence (selection) on phosphorylation then it would be predicted that one group would have a significantly different proportion of (potential) phosphorylation sites including a Met residue. At least superficially this is not the case. In the very limited data available to us, aerobes (e.g., *Escherichia coli *and *Staphylococcus aurius*) and anaerobes (e.g., *Bacillus fragilis *and *Clostridium botulinum*) have a similar proportion of sites with Met (Additional file 1). It would be particularly interesting to evaluate the P-proteome of an organism such as the nematode *Ascaris suum *which has both aerobic and anaerobic components of its' life-cycle. Unfortunately, no P-proteomic data are currently available.

While the regulatory importance of protein PTM has been long known, it has only been more recently that roles for multiple and interacting PTM's have been appreciated [77]. Examples range from interaction between multiple instances of the same PTM at different sites (e.g., prior or priming phosphorylation of site A is necessary for subsequent phosphorylation of site B [14-17]), through hierarchical responses to multiple PTM of the same site [5,37,46], or differential responses to multiple different PTM at different sites [78], and ultimately to crosstalk among different PTM at different sites [79]. A protein with 10 instances of a single PTM could give rise to 1024 (2^n^) different molecular species, but if there are two types of PTM there is the potential to generate 59049 (3^n^) species [46]. The enormity of this molecular diversity would generate a gradient in response that will allow an exquisitely fine control over metabolic activity, signalling, and cellular function [46].

Our initial interest in crosstalk between Ser/Thr/Tyr-phosphorylation and Met oxidation was stimulated by observations that two important metabolic enzymes could be responsive to cellular redox signalling through this mechanism [50-52]. While this study was underway, it came to our attention that there have been additional reports of what now appears to be crosstalk between phosphorylation and Met oxidation. Decreased phosphorylation is correlated with increased Met oxidation of α-synuclein in MetSO knockdown mutants of both yeast and mice [80,81]. Site-specific oxidation of Met in calmodulin was shown to affect its structure, and thus its interaction with Ca2+/calmodulin-dependent protein kinase II and subsequent downstream signalling [82]. There is also an analogous example [83] where activity of a protein P-Tyr-phosphatase is regulated by reversible Cys-oxidation. Finally, there is the intriguing recent report that Met oxidation in *A. thaliana *is responsive to cGMP [84].

Results from GO analysis indicated that the occurrence of Met near phosphorylation sites could be more prevalent in proteins related to signalling such as kinases and stress related proteins, and could be common to all three taxa - plants, yeast, and animals (Figure 4 and 6). Many phosphorylation motifs - kinase recognition signatures - contain hydrophobic Met residue [57,62]. Oxidation of Met to MetSO causes a shift from hydrophobic to hydrophilic in nature, and would thus likely disrupt kinase recognition [50]. So it is potentially useful for kinases and stress signalling proteins to have evolutionary selection for Met near phosphorylation sites for potential crosstalk. This would allow direct communication of oxidative signals to other mainstream processes.

## Conclusion

Our study identified a large number of phosphorylation sites which include a vicinal Met residue. The proteins containing these sites can potentially function as redox-sensors capable of transducing input from ROS signalling to regulation of phosphorylation. These observations should stimulate further research on PTM crosstalk and control of protein function in response to oxidative signalling.

## Competing interests

The authors declare that they have no competing interests.

## Authors' contributions

RSPR participated in design of the study and conducted the computational analyses. JJT and DX participated in design and coordination of the study. JAM conceived the study, participated in its design and coordination, and drafted the manuscript. All authors read and approved the final manuscript.

## Supplementary Material

Additional file 1 **S1.pdf**. Percentage of Ser/Thr/Tyr sites (Ser, Thr, and Tyr from top to bottom) with Met within a ± 6 window in different species. Click here for file

Additional file 2 **S2.pdf**. Distribution of other hydrophobic residues (Val/Ile/Leu/Phe, %) around non-Ser/Thr/Tyr and Ser/Thr/Tyr (individually from left to right, all and phosphorylated) sites in plant (A), yeast (B), animal (C), bacterial (D) and archaeal (E) P-proteins. Click here for file

Additional file 3 **S3.pdf**. Consensus sequences (13-mers) for phosphorylation sites with highly conserved Met (≥ 7 Met among eight taxa). Click here for file

## Declarations

Publication costs for this article were funded by the United Stated Department of Agriculture, Agricultural Research Service, Project number 3622-21000-032-00D.

This article has been published as part of *BMC Bioinformatics *Volume 14 Supplement 14, 2013: Proceedings of the Tenth Annual MCBIOS Conference. Discovery in a sea of data. The full contents of the supplement are available online at http://www.biomedcentral.com/bmcbioinformatics/supplements/14/S14.
