# Supplementary figures and images for: Circles within circles: crosstalk between protein Ser/Thr/Tyr-phosphorylation and Met oxidation
Source: BMC Bioinformatics. 2013 Oct 9;14(Suppl 14):S14. doi: 10.1186/1471-2105-14-S14-S14 (PMC3851202; doi:10.1186/1471-2105-14-S14-S14)

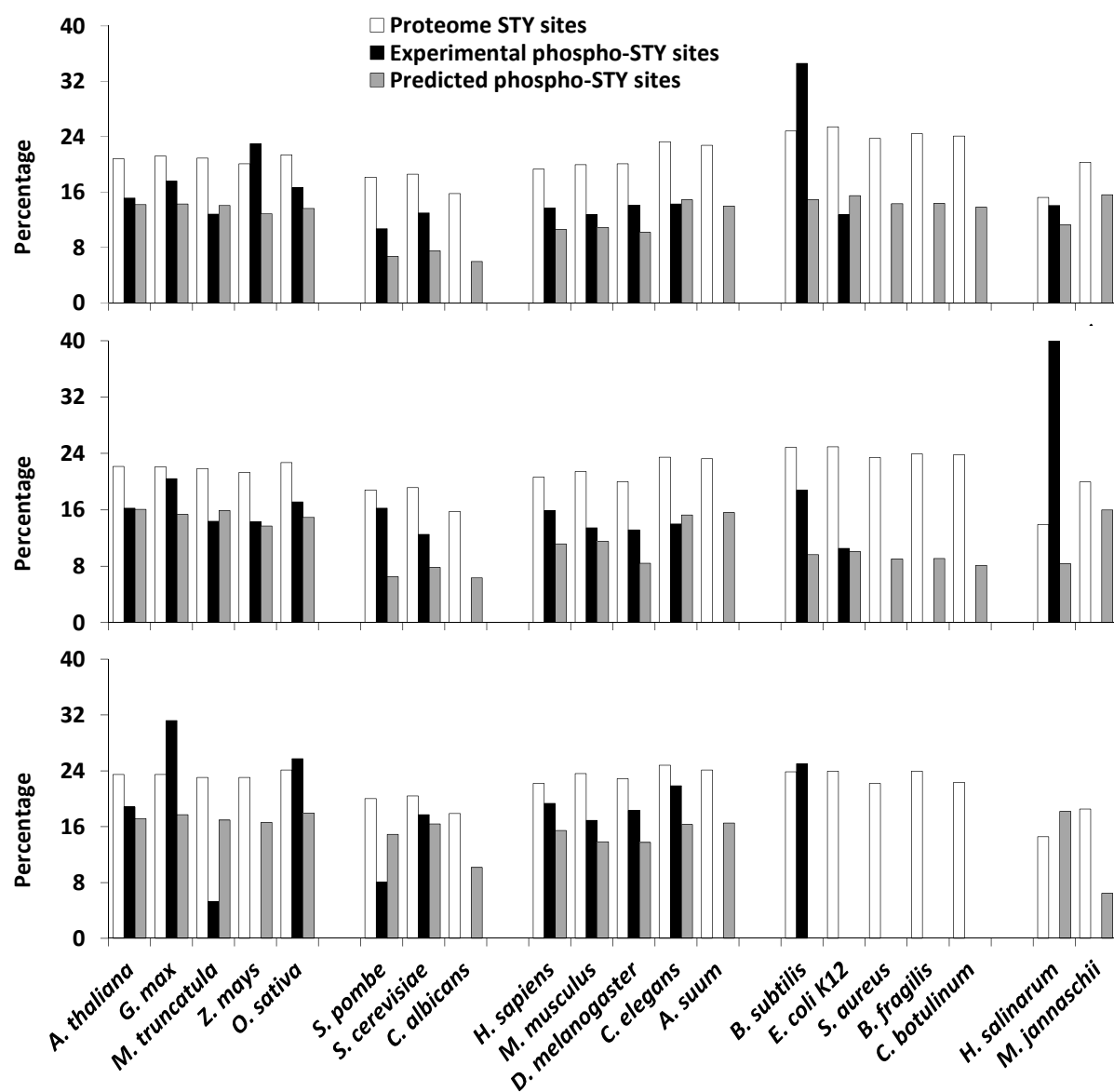

Supplement: Additional file 1 — S1.pdf. Percentage of Ser/Thr/Tyr sites (Ser, Thr, and Tyr from top to bottom) with Met within a ± 6 window in different species. [file 1471-2105-14-S14-S14-S1.pdf]

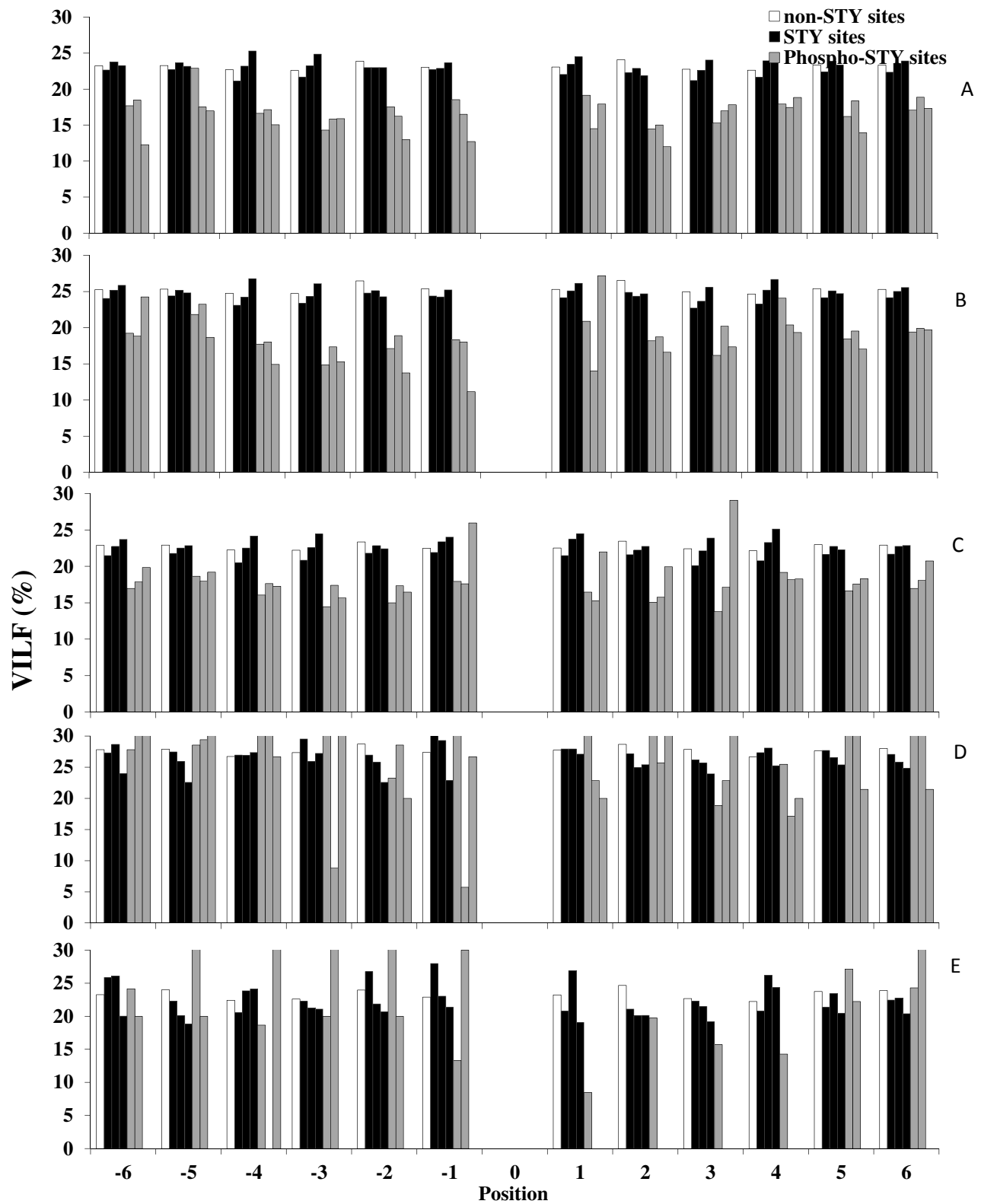

Supplement: Additional file 2 — S2.pdf. Distribution of other hydrophobic residues (Val/Ile/Leu/Phe, %) around non-Ser/Thr/Tyr and Ser/Thr/Tyr (individually from left to right, all and phosphorylated) sites in plant (A), yeast (B), animal (C), bacterial (D) and archaeal (E) P-proteins. [file 1471-2105-14-S14-S14-S2.pdf]
