# Supplementary material for: Circles within circles: crosstalk between protein Ser/Thr/Tyr-phosphorylation and Met oxidation
Source: BMC Bioinformatics. 2013 Oct 9;14(Suppl 14):S14. doi: 10.1186/1471-2105-14-S14-S14 (PMC3851202; doi:10.1186/1471-2105-14-S14-S14)
Supplement: Additional file 3 — S3.pdf. Consensus sequences (13-mers) for phosphorylation sites with highly conserved Met (≥ 7 Met among eight taxa). [file 1471-2105-14-S14-S14-S3.pdf]

Position	Consensus sequence	Protein name	Arabidopsis ID
+6Y	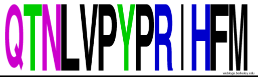	Tubulin alpha-3	AT5G19770.1
-6S	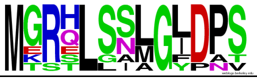	Nucleolar GTP binding protein	AT1G50920.1
-4	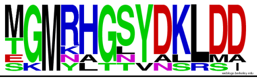	RNA polymerase	AT4G21710.1
-2	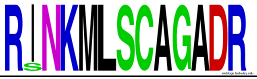	Senescence associated protein	AT1G66580.1
-2	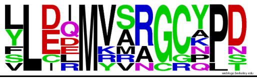	Pentatricopeptide repeat protein	AT1G09900.1
-6Y	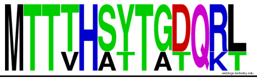	Glyceraldehyde 3 phosphate dehydrogenase A	AT3G26650.1
-3	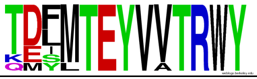	MAP kinase 13	AT1G07880.2
+3	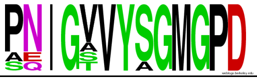	Ntn aminohydrolase	AT1G79210.1
+3	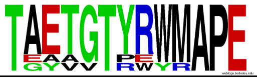	ACT-like protein tyrosine kinase	AT2G17700.1
-6T	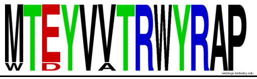	MAP kinase 6	AT2G43790.1
-6/+4	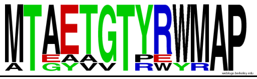	ACT-like protein tyrosine kinase	AT2G17700.1
-6	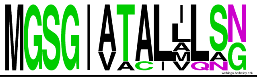	Enoyl-CoA hydrolase	AT4G29010.1
-5	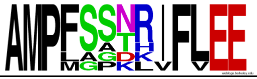	Lycopene cyclase	AT3G10230.1
-4/+6	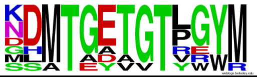	MAP kinase	AT5G50000.1
-4	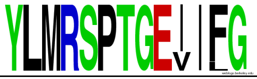	Photosystem II reaction center protein C	ATCG00280.1
-4	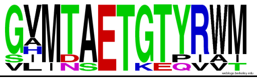	ACT-like protein tyrosine kinase	AT2G17700.1
-3	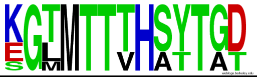	Glyceraldehyde 3 phosphate dehydrogenase A	AT3G26650.1
-3	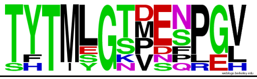	ATP binding microtubule motor protein	AT1G18550.1
-3	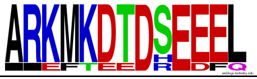	Calmodulin 7	AT3G43810.1
-3	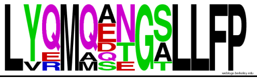	S-adenosyl homocysteine hydrolase	AT3G23810.1
-3	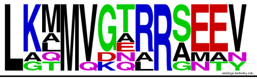	Protein kinase	AT2G42960.1
-3	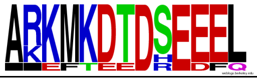	Calmodulin 4	AT1G66410.1

-2	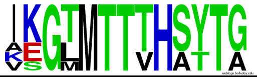	Glyceraldehyde 3 phosphate dehydrogenase A	AT3G26650.1
-2	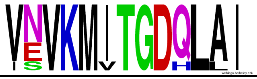	H+ ATPase 2	AT4G30190.1
-1	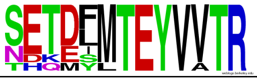	Protein kinase	AT1G07880.2
-1	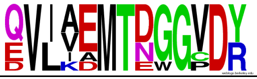	Alcohol dehydrogenase 1	AT1G77120.1
-1	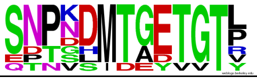	Protein kinase	AT5G50000.1
-1	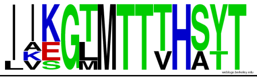	Glyceraldehyde 3 phosphate dehydrogenase A	AT3G26650.1
-1	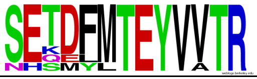	MAP kinase 6	AT2G43790.1
-1	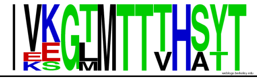	Glyceraldehyde 3 phosphate dehydrogenase B	AT1G42970.1
-1	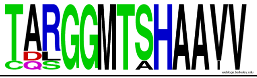	Pyruvate orthophosphate dikinase	AT4G15530.2
-1	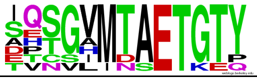	ACT-like protein tyrosine kinase	AT2G17700.1
-1	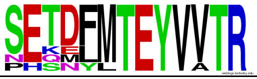	MAP kinase 10	AT3G59790.1
+1	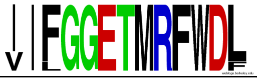	Photosystem II reaction center protein C	ATCG00280.1
+2	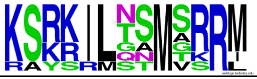	Vesicle transport V snare 13 protein	AT3G29100.1
+3	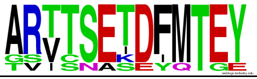	MAP kinase 6	AT2G43790.1
+4	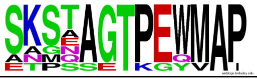	Protein kinase	AT1G08720.1
+5	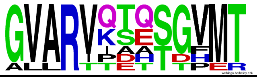	ACT-like protein tyrosine kinase	AT4G35780.1
+6	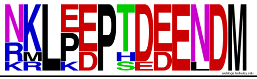	Mitochondrial ferredoxin 2	AT4G21090.2
-6S	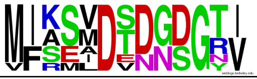	Calcium dependent protein kinase 21	AT4G04720.1
-6	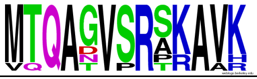	Nascent polypeptide associated complex alpha	AT3G49470.1
-6	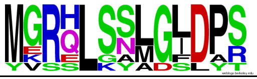	Nucleolar GTP binding protein	AT1G50920.1
-5	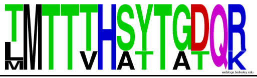	Glyceraldehyde 3 phosphate dehydrogenase A	AT3G26650.1
-5	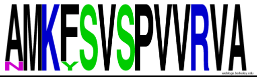	Elongation factor G	AT1G56070.1
-5	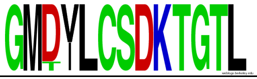	H+ ATPase 2	AT4G30190.1

-5	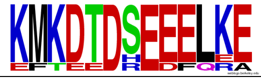	Calmodulin 7	AT3G43810.1
-5	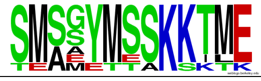	Heat shock protein 90	AT5G52640.1
-5	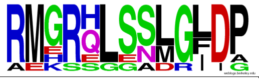	Nucleolar GTP binding protein	AT1G50920.1
-5	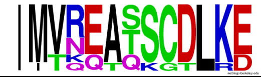	Ribosomal protein S3Ae	AT4G34670.1
-4	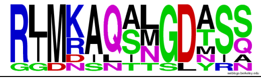	Heat shock protein 90	AT3G07770.1
-3	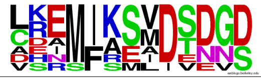	Calcium dependent protein kinase 21	AT4G04720.1
-3	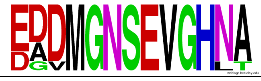	Phosphoglycerate mutase	AT1G09780.1
-3	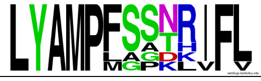	Lycopene cyclase	AT3G10230.1
-2	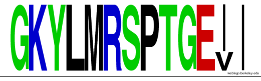	Photosystem II reaction center protein C	ATCG00280.1
-2	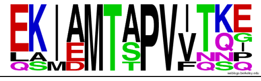	Heme binding protein	AT2G37970.1
-2	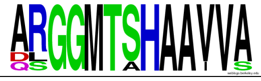	Pyruvate orthophosphate dikinase	AT4G15530.2
-2/+4	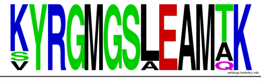	Aldolase	AT1G79470.1
+1	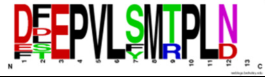	Photosystem II reaction center protein C	ATCG00280.1
+2	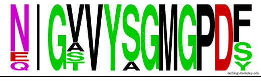	Ntn aminohydrolase	AT1G79210.1
+3	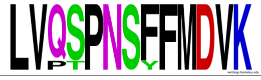	Ribosomal protein S27	AT3G61110.1
+3	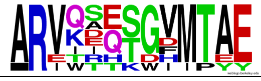	ACT-like protein tyrosine kinase	AT2G17700.1
+3	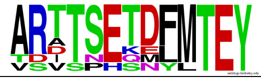	MAP kinase 10	AT3G59790.1
+3	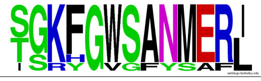	Heat shock protein 90	AT2G04030.1
+4	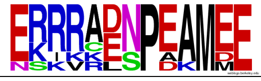	CDC48 protein	AT3G53230.1
+5	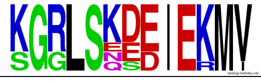	Heat shock protein 70B	AT1G16030.1
+6	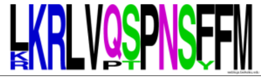	Ribosomal protein S27	AT3G61110.1
+6	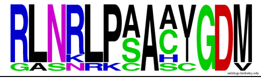	Ribosomal protein L14p-L23e	AT1G04480.1
+6	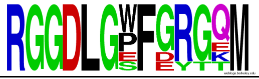	Peptidylprolyl cis-trans isomerase	AT2G18040.1

Protein names are based on *Arabidopsis*
